# Evaluation of safety standards accomplishment in educational hospitals of Neyshabur University of Medical Sciences-Iran

**DOI:** 10.1016/j.mex.2019.02.034

**Published:** 2019-03-05

**Authors:** Ali Kavosi, Mehrdad Rohaninasab, Sara Shirdelzadeh, Gholamreza Mohammadi, Ali Movahedi, Hossein Nasiri, Akram Sadat Montazeri, Mohammadreza Aryaeefar, Mona Hosseini, Ali Akbar Mohammadi

**Affiliations:** a Nursing Research Center, Faculty Member, Golestan University of Medical Sciences, Gorgan, Iran; b Student Research Committee, Neyshabur University of Medical Sciences, Neyshabur, Iran; c Department of Nursing, Faculty Member of Neyshabur University of Medical Sciences, Neyshabur, Iran; d Department of Anesthesia and Operating Room, Faculty Member of Neyshabur University of Medical Sciences, Neyshabur, Iran; e Department of Environmental Health Engineering, School of Health, Isfahan University of Medical Sciences, Isfahan, Iran; f Department of Environmental Health Engineering, Neyshabur University of Medical Sciences, Neyshabur, Iran

**Keywords:** Standards, Hospitals, Safety, Neyshabur

## Abstract

Patient’s safety and staff in hospital is one issue that has always been considered as an important principle by experts in health systems. Therefore, for increase safety, standards and safety regulations must be considered. This study aims to evaluation of safety standards accomplishment in educational hospitals of Neyshabur University of medical Sciences. This cross-sectional descriptive study, safety standard status of all wards including 17 various wards from 22 Bahman hospital and 14 various wards from hakim Hospital in Neyshabur city (2016) was investigated. Data were collected using a questionnaire safety standard status hospitals (264 questions). Also data analyzed by SPSS16 software, using descriptive (Mean ± SD) and inferential statistics (*T*-Test). The results revealed that Safety standard status in 22bahman and hakim hospital were weak (2.42 ± 0.14) and moderate (3.04 ± 0.18) respectively. Also result showed in Hakim hospital, the highest and lowest safety standard status in Internal and Administrative-financial unit was (3.42 ± 0.19) (1.36 ± 0.58) respectively. In addition in hakim hospital, the highest and lowest safety standard status in operation room and administrative-financial unit (3.53 ± 0.28), 1.36 ± 0.58) respectively. According to the result, the safety condition in hospitals of Neyshabur city is moderate and poor status. However, imperfect implementation of safety protocols can endanger safety conditions in hospital. Therefore, it is necessary to take the required correcting measures to ensure full safety in hospitals.

• Safety is defined by development of systems for preventing incidents, injuries, and other unpleasant events in organizations.
• This article showed the safety status is not appropriate in most units of hospitals, which can lead to dangers for patients and personnel.
• It is suggested for future studies to compare the safety status of different provinces.

## Graphical abstract

**Figure:**
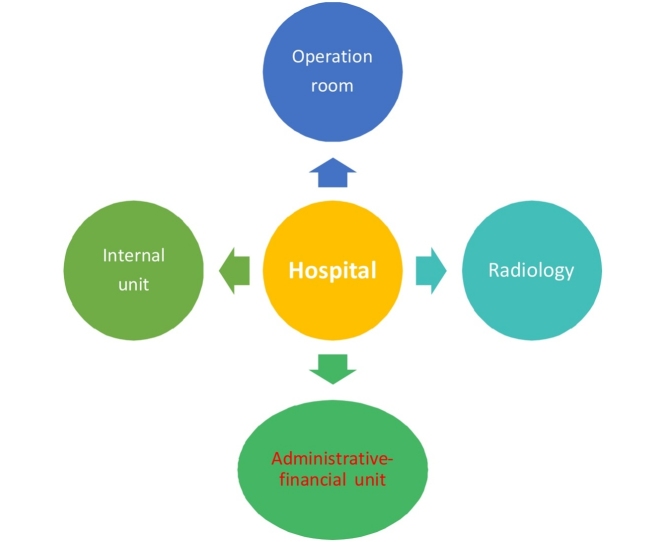

**Specifications Table**

**Table:** 

**Subject area:**	Environmental Science
**More specific subject area:**	Safety Standards Accomplishment in Educational Hospitals
**Methods name:**	Evaluation of Safety Standards Accomplishment
**Name and reference of original method:**	Dückers M, Faber M, Cruijsberg J, Grol R, Schoonhoven L, Wensing M. Safety and risk management in hospitals: Health Foundation; 2009.
**Resource availability:**	The data are available with this article.

## Method details

Safety is defined by development of systems for preventing incidents, injuries, and other unpleasant events in organizations [1]. At a glance, hospital’s environment looks clean and safe; but, there are some threats for the existence of hospitals such as various tasks, and different dangers and incidents including electrocution, explosion, fire, chemical substances, and nosocomial infections which all sensitize managers for protection of human and financial resources [2]. Therefore, by developing standards and great progresses in this field, the main goals of managers become saving in human resources, materials, energy, consumer support, safety, health, natural environment, and better communication [3]. Therefore, hospitals which are playing important roles in health promotion, should provide standards for better quality management. Actually, hospital’s standards are one of valuable conceptual elements of organization because of their wonderful role in declaring the expected functions and assisting in task evaluation. Nowadays, hospitals are moving towards quality improvement to lead all hospital quality standards to insuring personnel and patients; so, one of the most important standard is providing a safe and healthy environment for patients and personnel [4,5]. On the other hand, the hospital's safety standard (HSS) is one of modern management principal in medicine, and it is important economically and ethically. Eventually, accomplishing safety standards (SS) in hospital environment will result in improvement of efficacy, effectiveness, and consequently efficiency [6]. Ignoring HSS can cause irreparable damage to financial resources and lives, which may be sometimes impossible to compensate. Therefore, ensuring of providing qualified and safe services, one of the main task of healthcare system, is a priority and absolutely appropriate policy making, planning, implementing and controlling of it needs special attention and consideration [7].

Experiences showed work related injuries is more in healthcare centers rather than industries. So, hospital personnel must make double effort for self-protection and providing safe environment for patients [8]. All countries stated the features and requirements of organizational safety in their special laws and some in their constitution, which is showing its sensitivity and importance [9]. Behnoodi showed general and specialized hospitals had used the standards the same and international criteria and principals approximately accomplished in the field of organization and facilities, but not in the field of equipment, and designing and usage of sanitary principals [10]. Based on a study in U.S., failure to accomplish principals can increase the mean of hospital’s cost 400 $ per patient; also, in another study on safety in England, Hosper clarified implementation of a safety program for 7 years, saved more than 5 million pounds in cost of hospitals, which was due to the 30% decrease of safety costs in the participating units [11]. Obviously, the most effective, appropriate, and lowest cost method of fighting risks in hospitals is prevention of their occurrence (by providing and preserving SS in units) [12].There are numerous study on safety and evaluation of standards of different units in hospitals and healthcare centers such as Horighuchi [13], Finnegan [14], Prasad [15], Danne [16], Homayoonnejad [17], Alizade [18], Ayoubian [19], Mosavi [20], and Amiri [21]; most of them focused on assessment of a specific unit and feature, and also there is no study on SS in Neyshabur city. Therefore, this study designed for assessing the rate of safety standards accomplishment in educational hospitals of Neyshabur to assist improvement of safety level and decreasing the probability of dangers and incidents.

## Methodology

This descriptive cross-sectional study was done in 2016 and the population was consisted of all units of educational hospitals of Neyshabur University of Medical Sciences (17 units of 22 Bahman hospital and 14 units of Hakim hospital). Data were gathered by the hospital safety standards questionnaire which contains 264 questions including general features of hospital, total safety standards of hospital, service, laboratory, installation, radiology, operation room, central sterile room, inpatient wards (internal, surgical, heart, and ICU, CCU), financial and administrative units, and emergency ward [22]. Each question has 6 choices and score from 0 to 5 (0: never, 1: very low, 2: low, 3: moderate, 4: much, 5: too much). Total safety standard score calculated by dividing total scores of all question by 264 and Mean total would be between 0–5; the score of 4.26–5 shows the SS status is very good, 3.51–4.25 good, 2.75–3.50 moderate, 2.01–2.75 poor, 1.25–2 very poor, and less than 1.25 is unacceptable [22]. The reliability and validity of this questionnaire is evaluated by Mosadegh et al. that Cronbach’s alpha was investigated (α = 0.93) [22].

Researcher fulfilled the questionnaire by observing different units of hospitals and their documents, and also by interview with manager’s hospitals, hospital safety manager, and units’ managers. Data were analyzed by SPSS 16 and descriptive (Mean ± SD) and inferential statistics (*T*-Test). The significance level was 0.05. Ethical issues of research were considered through obtaining the permission of research chancellor of Neyshabur University of Medical Sciences and total results fed back to hospitals while keeping confidentiality.

## Results

Results showed the best safety standards status (SSS) of the 22Bahman hospital belonged to the Cardiac Care Unit (CCU), and internal ward and the lowest SSS belonged to radiology, CSR, and financial-administrative units. Hakim hospital had the best SSS in operation room, radiology and internal ward, and the lowest SSS in laundry and financial-administrative units. Also there was significant difference between some of the same units of 2 hospitals (p < 0.05) (Table 1).

**Table 1:** Comparison of the Mean and SD of SSS in same units of two hospitals.

22Bahman Hospital		Hakim hospital		*T*-Test
Unit	Mean ± SD	Unit	Mean ± SD	
Emergency	2.74 ± 0.27	Emergency	3.13 ± 0.24	P = 0.091
Internal	3.42 ± 0.19	Internal	3.23 ± 0.16	P = 0.437
Operation room	2.82 ± 0.41	Operation room	3.53 ± 0.28	P = 0.001
Radiology	1.71 ± 0.87	Radiology	3.43 ± 0.11	P = 0.001
ICU	2.19 ± 0.14	ICU	2.76 ± 0.31	P = 0.104
Clinic	2.09 ± 0.39	Clinic	2.86 ± 0.32	P = 0.091
CSR	1.79 ± 0.54	CSR	2.51 ± 0.44	P = 0.001
Laboratory	2.27 ± 0.42	Laboratory	2.87 ± 0.21	P = 0.192
Service/Installation	2.63 ± 0.51	Service/Installation	2.46 ± 0.63	P = 0.611
Financial-administrative	1.36 ± 0.58	Financial-administrative	1.36 ± 0.58	P = 1
Surgical (female)	2.61 ± 0.47	Surgical (female)	2.95 ± 0.37	P = 0.382
Laundry	2.21 ± 0.43	Laundry	2.09 ± 0.63	P = 0.268
Warehouse	2.59 ± 0.33	Warehouse	2.97 ± 0.13	P = 0.691
Kitchen	2.68 ± 0.21	Kitchen	2.84 ± 0.49	P = 0.734
Surgical (male)	2.47 ± 0.34	–	–	–
Chemotherapy	2.33 ± 0.37	–	–	–
CCU	3.38 ± 0.29	–	–	–

Findings revealed the total SSS in 22Bahman and Hakim hospitals were poor (2.42 ± 0.14) and moderate (3.04 ± 0.18), respectively (Table 2).

**Table 2:** Mean and SD of Total SSS in educational hospitals of Neyshabur.

Hospital	Mean ± SD of total SSS	SSS
22Bahman	2.42 ± 0.14	Poor
Hakim	3.04 ± 0.18	Moderate

## Discussion

In recent century, known as scientifically and industrial advanced technologies and fast exchange of information, international healthcare organizations pay attention to safety and prevention of incidents, promoting human resources’ health, and keep equipment out of damage. Diagnostic and therapeutic units are the most important units of each hospital and need special attention because of their complex and expensive equipment and technologies, and also expert personnel [23]. Therefore, this study was designed to assess the SSS of two educational hospitals of Neyshabur.

Our findings revealed the SSS in 22Bahman hospitals and Hakim hospitals Neyshabur city were respectively poor (2.42 ± 0.14) to moderate (3.04 ± 0.18); which means none of them is in appropriate status. A study conducted by Fathi et al. in Kurdestan University of Medical Sciences their result showed one of educational hospital had good SSS, 30% were in poor and the rest of them in moderate status [24]. Another research done by Kakavand et al., on assessment of Safety Condition in One of the Teaching Hospitals in Kermanshah, result proved that 66% hospital’s units of Kermanshah had very poor status and the other were in moderate status [25]. In addition a study in Shiraz, result showed the SSS of hospitals were poor, especially in safety management and the plan for emergency conditions [26]. Jahani et al. revealed the moderate status of safety management in hospitals of Babol University of Medical Sciences [27]. Another study declared the inappropriate status of safety in most units of Kerman's hospitals [9]. According to importance of safety and sanitary in improving the effectiveness, efficacy and eventually efficiency of hospitals, it is essential to pay special attention to safety standards accomplishment.

The SSS of radiology units were inappropriate and in one hospital had the lowest score, and statistical analysis showed the significance difference between radiology units of 2 hospitals. Khalooei et al. declared the lowest score belonged to radiology unit [9]. An assessment of radiology units of Isfahan in 2009, had reported poor to moderate SSS of them [28]. Most problems of our radiology unit included absence of a specific guideline for classification and evacuation of patients in case of incidents, not exposing the safety guideline for the personnel, not available first aid means, absence of fire detection system, and no planned program for safety control of available devices. In this study, the SSS of ICU wards and emergency ward were poor, except the CCU of 22Bahman hospital which had moderate. In similar studies had done in Kerman and Kermanshah cities, poor and inappropriate status were reported for emergency wards [9,29]. Also, Moeini et al. found out the standards of special wards of Arak's hospitals were far from reliable standards, which is compatible with our results [30]. Yavari et al. showed the special care units of some hospitals in Tehran had appropriate SSS and some had not [31]. But incompatible with ours, a study in Isfahan had showed the appropriate SSS in special care units, particularly in army’s hospital [19]. This difference could be due to the place, time, and assessment scale and management policy of hospitals. It should be noted that the main problems of our emergency ward included the absence of another fast accessibility to emergency ward except the main door of hospital, personnel’s lack of knowledge about safety standards of unit, lack of resuscitation facilities and equipment; in special care units the main safety problems were lack of fire detection and fight system, absence of first aid means for personnel, no emergency exit, and no regular evaluation of electrical and gas equipment. These wards require appropriate safety status, according to crucial and sometimes life-threatening conditions of patients in these wards, the anxiety of such hard conditions for both patients and their families, and the need for fast treatment.

Our results revealed the SSS in inpatient wards both hospitals were moderate. A study in Kerman showed the appropriate SSS of inpatients wards [9]. But, in another study in Kermanshah the SSS of inpatient wards were poor and moderate [25]. Chatrooz et al. showed the appropriate status of inpatient wards in some hospitals of Tehran [32], which is not compatible with our results. The main problems of our inpatient wards were failure to announce and impart specific codes for unexpected incidents, personnel lack of knowledge about safety standards, and lack of their participation in regular practices, no emergency exit, and lack of fire detection and fight system.

The mean of SSS in laboratory units, which were poor in both hospitals, demonstrated that the improvement of safety status needs various implementations. Accomplishing safety standards in laboratories is more important than other units because of processes like direct contact with samples, samples’ preparation methods, and their culture in laboratory environment; thus, it is essential to use safety standards in building design, and equipment application, and also personnel must apply them [33]. Khalooei et al. showed the SSS of laboratory were inappropriate [9]. 3/4 of labs in Kurdestan had inappropriate safety status [24]. The main problems of labs in our study included no regular evaluation of equipment, electrical means and cables, no participation of personnel in classes of unexpected incidents preparation, lack of first aid means, and lack of fire detection and fight system.

Our results revealed the moderate to good SSS in operation rooms of both hospitals; although, there was significant difference between them. Mosavi et al. reported the operation rooms in Tehran had 89.9% safety [20]. Gazerani et al. declared the good SSS of Bojnourd hospitals’ operation rooms [34]. Foji et al. found the SSS of 3Sabzevar’s hospitals appropriate [35]. Also, another study in Shiraz by Mohebati et al. detected the SSS accomplishment in operation rooms were more than 50% [36]. Operation room as the heart of hospital receive more attention for safety standards accomplishment in dimensions of building standards, fire fight, personnel and patient safety, and infection control in order to gain more score in evaluating hospitals. Thus, it may be the reason of its appropriate SSS.

Supportive units such as CSR, laundry, kitchen, warehouse, and installation play important role in safety of personnel and patients, and also in prevention of incidents. In our study their SSS were poor which is incompatible with Mosavi et al. [37], and Chatroozi et al. [32], but compatible with Kakvand et al. [25] in Kermanshah. Firefighting was the major problem of these units because of the old building of Neyshabur’s hospitals; although, it is the most important challenge of designers and users of healthcare centers. Annually 8000 fire occur in hospitals, national firefighting has reported [38].

Finally, the knowledge of personnel about safety standards and readiness for unexpected incidents were low in Neyshabur’s hospitals and need to be educated. Numerous studies on hospital safety emphasis on the importance of personnel knowledge and its effect on improving the safety status; which can explain the appropriateness of SSS in mentioned studies [39]. Evidenced showed personnel knowledge of safety can result in decreasing hospital's incidents; therefore, it is essential that hospital's manager and safety committee take action in this regard [40]. The appropriate safety standards in studied hospitals recited the good knowledge of personnel which is gained by the activity of safety committee. Hence one of hospital managers’ responsibility and healthcare centers tasks is to provide safe environment for patients and personnel, managers with cooperation of safety committee should develop safety programs for fixing probable defects, incidents prevention, and appropriate function in case of danger [41]. Furthermore, success of any program in organization need the cooperation and support of its leader; in absence of this support, no program will succeed. Other influencing factors on success or defeat of a safety program include open work communication, an error-reporting system, and existence of a safety culture in organization [40].

We had some limitations such as our cross-sectional method, the absence of comprehensive studies on all units of hospitals in Iran which limits the comparison of results, and the endeavor of managers of units to demonstrate status better than reality.

## Conclusion

This article showed the safety status is not appropriate in most units of hospitals, which can lead to dangers for patients and personnel. Therefore, some implementations is essential like educating personnel and mangers, set and accurate implementation of laws and principals, regular and purposive supervision on safety, and considering the safety principal accomplishment as a part of hospital evaluation and grading. It is suggested for future studies to compare the safety status of different provinces.

## Conflict of interest

The authors of this article declare that they have no conflict of interests.
